# Temporal changes in arbuscular mycorrhizal fungi communities and their driving factors in *Xanthoceras sorbifolium* plantations

**DOI:** 10.3389/fmicb.2025.1579868

**Published:** 2025-05-29

**Authors:** Yuexin Zhang, Yunxia Ma, Xiuzhi Ma, Cuiwei Li

**Affiliations:** ^1^College of Forestry, Inner Mongolia Agricultural University, Hohhot, China; ^2^College of Desert Control Science and Engineering, Inner Mongolia Agricultural University, Hohhot, China

**Keywords:** *Xanthoceras sorbifolium*, stand age, forest management, arbuscular mycorrhizal fungi, arbuscular mycorrhizal fungi community

## Abstract

Arbuscular mycorrhizal fungi (AMF) communities are influenced by soil nutrients and plant and litter traits during forest ecosystem development. However, the extent to which these factors influence AMF communities in *Xanthoceras sorbifolium* plantations is unclear. In this study, rhizosphere soil samples were collected from 5-, 13-, 24-, 35-, 47-, and 56-year-old *X. sorbifolium* plantations. The AMF community was analyzed using Illumina MiSeq sequencing, and AMF spores were isolated and identified by wet sieving. The results showed that *X. sorbifolium* can establish a symbiotic relationship with AMF at different forest ages. In total, 5,876 AMF amplicon sequence variant (ASVs) were obtained from the soil samples and classified into 1 phylum, 4 classes, 6 orders, 12 families, and 15 genera. *Glomus* was the dominant genus. In addition, the diversity of AMF communities increased and then decreased with the age of *X. sorbifolium*, with no significant changes observed between 35-, 47-, and 56-year-old plantations. AMF community variance was primarily determined by soil-specific factors, with soil pH and root C content being the most influential. The results revealed the factors that drive AMF communities during the development of *X. sorbifolium* and provide valuable information for future conservation and planting management.

## 1 Introduction

Mycorrhizal symbiosis is a common form of mutually beneficial relationship between fungi and plants in nature. Arbuscular mycorrhiza (AM) is a type of mycorrhiza, which is formed by the symbiosis between arbuscular mycorrhiza fungi (AMF) and host plants (Fei et al., 2022). AMF are non-specific beneficial microorganisms that can establish a symbiotic relationship with most higher terrestrial plants; thus, they are important components of natural ecosystems. AMF have an important role in the formation of stable soil aggregates, soil carbon and nitrogen cycling processes, and plant community succession ecological processes, with potentially valuable implications for sustainable ecosystems (Gui et al., 2017; Mohammadi et al., 2019). Changes in the structure and diversity of AMF communities affect plant performance and ecosystem stability (Wagg et al., 2011) and improve plant growth by increasing nutrient intake (Shao et al., 2021). Numerous studies have demonstrated that AMF communities are closely related to external environmental conditions such as soil physicochemical properties, vegetation type and altitude (Liu et al., 2023). However, for a single target plant, the age of the stand may be an important determinant affecting the AMF community (Zhang et al., 2022; Pereira et al., 2014).

During plant growth, tissues and organs such as leaves and root systems interact with each other and jointly regulate the functional traits of plants. Leaves, as an important organ for plant photosynthesis, have elemental contents that not only indicate the nutrient supply capacity of the soil but also characterize the response and adaptation to environmental changes (Wright et al., 2001). The plant root system is an important organ connecting the plant and the soil, and fine roots, as the most sensitive and active part of the root system, are an important source of soil nutrient pools, with the total global fine root C pool being more than 5% of the atmospheric C pool. Moreover, the fine roots are the main organ by which plants expand the soil space, thereby shaping the physical environment of the soil and allowing the transport of nutrients and C elements to the surrounding microorganisms (Nadelhoffer, 2000). The morphology and nutrient content of plant leaves and root systems change as the stand develops (Vergutz et al., 2012), and plants can adjust their resource acquisition strategies by adjusting the changes in leaves and root systems to adapt to the eco-physiological processes of the tree during development. AMF are closely related to plant leaf and root traits (Chen et al., 2022; Kong et al., 2016), and plants can influence soil AMF communities at a regional scale by providing different quantities and qualities of litter and root inputs to AMF communities (Korenblum et al., 2020). As the stand age increases, changes in stand structure and tree biomass directly affect litter quality and decomposition rates; moreover, changes in litter traits can alter nutrient availability, fundamentally affecting AMF communities (Li et al., 2019). Soil physicochemical conditions can indirectly influence forest development through plant function and changes in litter traits. Soil nutrients, physical structure, and pH have been shown in a large body of literature to have important effects on AMF communities (Bai et al., 2022; Bonfim et al., 2016). Thus, AMF communities may be determined by interactions among soil, plant, and litter traits, and these interactions may be related to stand development. Although the effects of stand development on AMF community composition and diversity have been noted (Bennett and Groten, 2022; Zhu et al., 2024), the effects of relevant factors on AMF community composition and diversity during stand development require further investigation.

*Xanthoceras sorbifolium* is a rare woody oil tree species endemic to northern China that can be used as biodiesel feedstock. It has strong ecological adaptability and resistance to adversity and is an excellent tree species for wind and sand control, soil and water conservation, and desertification control (Wang et al., 2021). As the national energy strategy changes, the *X. sorbifolium* industry has received increasing attention and the area of artificial planting has been expanding. Thus, large areas of *X. sorbifolium*-producing areas have been established in Ningxia and Inner Mongolia in China, and the mode of operation is mainly pure forest (Xie et al., 2010). However, pure forests are prone to soil degradation, community decline, and reduced productivity (Wang, 2023), which ultimately limit the sustainable management of plantation forests and the ecological benefits of vegetation restoration. The *X. sorbifolium* industry is in its infancy, and current research is mainly focused on medicinal value, nutrient composition, and breeding for rapid propagation (Lang et al., 2020; Ji et al., 2020). Moreover, studies on *X. sorbifolium* mycorrhizal material are extremely limited. Zhu et al. (2015) found that the diversity of the rhizosphere fungal community of *X. sorbifolium* in different forest ages (5–10 a) was significantly correlated with soil environmental factors. Scholars have also identified Vesicular-Arbuscular (VA) mycorrhizal fungal structures in 10–12 a *X. sorbifolium* root systems (Zheng, 2017). Compared to the singularity of *X. sorbifolium* mycorrhizal studies by previous scholars, the present study was conducted in Wengniute Banner, Chifeng City, Inner Mongolia Autonomous Region, China, where *X. sorbifolium* plantation forests of different forest ages (5, 13, 24, 35, 47, and 56 years old) were selected, and AMF leaf blades, roots, litter, and rhizosphere soils were collected from AMFs of different forest ages. The objectives of this study were (1) to elucidate the patterns of changes in AMF community diversity, soil physicochemical properties, plant and litter characteristics with stand age in *X. sorbifolium* plantation forests. (2) Quantified the relative contributions of soil, plant, and litter properties and explored key drivers affecting AMF community change during *X. sorbifolium* development. A good understanding of the differences in soil microbial community composition and diversity in different stand stages of *X. sorbifolium* plantation forests, and in particular the magnitude of structuring effects, driven by changes in plant, root, or soil properties, which can provided reference for us to devise management strategies that regulate below-ground organisms in order to improve the nutrient sustainability of low-productivity *X. sorbifolium* plantation forests.

## 2 Materials and methods

### 2.1 Study area

**FIGURE 1:**
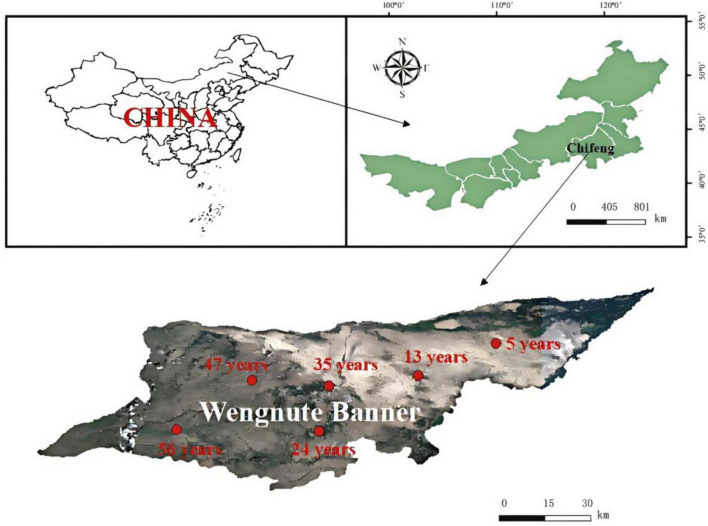
Location and sampling sites of forest farms in Udan Township, Wengniute Banner, Chifeng City, Inner Mongolia Autonomous Region, China.

This study was conducted at a forest farm in Wudan Town (119°45′48″-120°43′58″E, 42°27′26″-42°38′33″N) (Figure 1), Wengnute Banner, Chifeng City, Inner Mongolia Autonomous Region, China. The area has a typical temperate continental climate, with an average annual temperature of 5.9°C, average annual precipitation of 300–330 mm, and a soil type of mainly sandy chestnut-calcium soil.

### 2.2 Experimental design and plant and soil sampling

*X. sorbifolium* began to develop in the early 1960s and was planted on a large scale in the 1970s (Feng et al., 2022). The largest area of *X. sorbifolium* plantation forests in China was created in the study region, and the ideal chronological order of *X. sorbifolium* plantation forests with different developmental years was established, with the oldest being 56a. In August 2023, 5, 13, 24, 35, 47, and 56 years *X. sorbifolium* plantations (abbreviated as YF, MAF, NMF, MFI, MFII, and OMF, respectively) with similar stand conditions were selected. Three sample plots of 20 × 20 m were established in each stand, and the information of the sample plots is shown in Supplementary Table 1. In each sample plot, three *X. sorbifolium* with similar diameter at breast height (DBH) were randomly selected, and leaves, litter, rhizosphere soil, and fine roots (≤ 2 mm in diameter) were collected from each tree (see the Supplementary file for details on the sampling and measurement methods). Statistical results of plant and litter traits and soil properties are presented in Supplementary Tables 3–5, and specific variables and abbreviations are presented in Supplementary Table 2. All samples were taken back to the laboratory in a 4°C sampling box. The soil was divided into three portions, with one portion stored in a refrigerator at −80°C for soil DNA analysis, another portion of fresh soil was stored at 4°C for nitrate nitrogen (N-NO_3_^–^) and ammonium nitrogen (N-NH_4_^+^) analysis, and the remaining soil air-dried and passed through a 2 mm sieve for chemical analysis.

### 2.3 AMF colonization rate

The roots were cut into 1 cm pieces, decolorized in a KOH solution at 90°C for 60 min, and then rinsed. These root samples were softened with alkaline H_2_O_2_ (the softening time was adjusted according to the hardness of the roots), placed in 1% HCI solution for acidification, stained with Trypan blue dye solution containing 0.12% (w/v) at 80°C for 30 min, decolorized in lactic acid glycerol solution, washed with distilled water, sampled for microscopic examination, and photographed using an optical microscope (XSP-17C, ZSISS, Shanghai, China). Finally, the samples were observed and counted using the grid crossover method (McGonigle et al., 1990), and the AMF colonization rate was calculated. The AMF colonization rate (%) was calculated as the number of colonized root segments divided by the total number of tested root segments.

### 2.4 AMF spore identification

AMF spores were isolated using the wet sieve decantation-sucrose centrifugation method (Ianson and Allen, 1986). The morphology, color, and other characteristics of the AMF spores were observed using a microscope (XSP-17C, ZSISS, Shanghai, China), and descriptions and photographs for each species were obtained from the “Manual of Identification of Mycorrhizal Fungi of VA” (Wilson et al., 1983) and the International Center for the Preservation of Arbuscular Mycorrhizal Fungi (INVAM).^1^ The spore density (SD), separation frequency (F), and relative abundance (RA) of AMF spores were also determined at each sampling site. The formulas for these indicators are as follows:


(1) SD=total⁢number⁢of⁢AMF⁢spores⁢in⁢the⁢soil/soil⁢mass⁢(g)

×100%

(2) F=occurrence⁢frequency⁢of⁢a⁢certain⁢species/total

sample⁢number×100%

(3) RA=spore⁢number⁢of⁢a⁢certain⁢species/total⁢quantity⁢of

AMF⁢spores×100%

(4) Importance⁢value⁢(IV)=(F+RA)/2×100%

### 2.5 DNA extraction and Illumina MiSeq

DNA was extracted using a Soil DNA Kit (M5635-02; Omega Bio-Tek, Norcross, GA, United States). The quantity and quality of DNA were measured using a NanoDrop NC2000 spectrophotometer (Thermo Fisher Scientific, Waltham, MA, United States). The following PCR amplification primers were used to amplify the DNA samples: forward AMV4.5NF (5′-AAGCTCGTAGTTGAATTTCG-3′) and reverse AMDGR (5′-CCCAACTATCCCTATTAATCAT-3′). The PCR mixture consisted of 5 μl buffer (5 × ), 0.25 μl Fast pfu DNA polymerase (5 U/μl), 2 μl (2.5 mM) dNTPs, 1 μl (10 μM) forward and reverse primers, 1 μl DNA template, and 14.75 μl ddH_2_O. Thermal cycling began with an initial denaturation at 98°C for 5 min; followed by 25 cycles of 98°C for 30 s, 53°C for 30 s, and 72°C for 45 s; and a final cycle of 72°C for 5 min. The amplification products were subjected to 2% agarose gel electrophoresis, the target fragments were recovered using an Axygen Gel Recovery Kit (Thermo Scientific, Waltham, MA, United States), and paired-end sequencing was performed using an Illumina Miseq PE300 platform (Meiji Biomedical Technology Co., Shanghai, China).

Data de-duplication and quality filtering of the raw sequences were performed using FLASH (version 1.2.11) (Mago and Salzberg, 2011) and Fastp (version 0.20.0) (Chen et al., 2018). The sequences were then subjected to noise reduction and chimera removal using DADA2 in QIIME2 software (version 2019.4) to obtain Amplicon sequence variants (ASVs) (Callahan et al., 2016). The RDA Classifier Bayesian algorithm (version 2.11) was used to annotate the ASV taxonomy compared to the Maarj AM database, with a confidence threshold of 70%, and the community composition of each sample was counted at different species classification levels. The α-diversity index was calculated using the software QIIME2 (version 2019.4) (Bolyen et al., 2019).

### 2.6 Data analysis

One-way analysis of variance (ANOVA) was performed to assess the AMF spore density, mycorrhizal colonization rate, soil physical and chemical properties, and plant and litter characteristics of *X. sorbifolium*. The significance of differences was tested via multiple comparisons using the least significant difference (LSD) method. The similarity of AMF communities in *X. sorbifolium* plantations of different ages was evaluated using non-metric multidimensional scaling (NMDS) analyses based on Bray-Curtis distance calculations. Pearson’s correlation coefficient was used to analyze the relationships between the relative abundance of AMF genera and soil nutrients, plant characteristics, and apomictic traits. Variance partitioning analyses (VPA) were conducted using the vegan package in R to quantify the independent contributions of soil, plant, and litter traits to AMF community structure and their interactions. To avoid multicollinearity, only variables with a variance inflation factor (VIF) less than 10 were included. Redundancy analysis (RDA) was performed on the selected variables in the VPA (Supplementary Table 7) using R 4.22 software to assess the relationship between soil, plant, and litter traits and AMF communities. Detrended correspondence analysis (DCA) of AMF species data prior to RDA indicated that RDA was more appropriate for inferring relationships between AMF communities and environmental factors. Monte Carlo permutation tests were used to identify significant environmental factors affecting AMF communities.

## 3 Results

### 3.1 AMF colonization and spores

**FIGURE 2:**
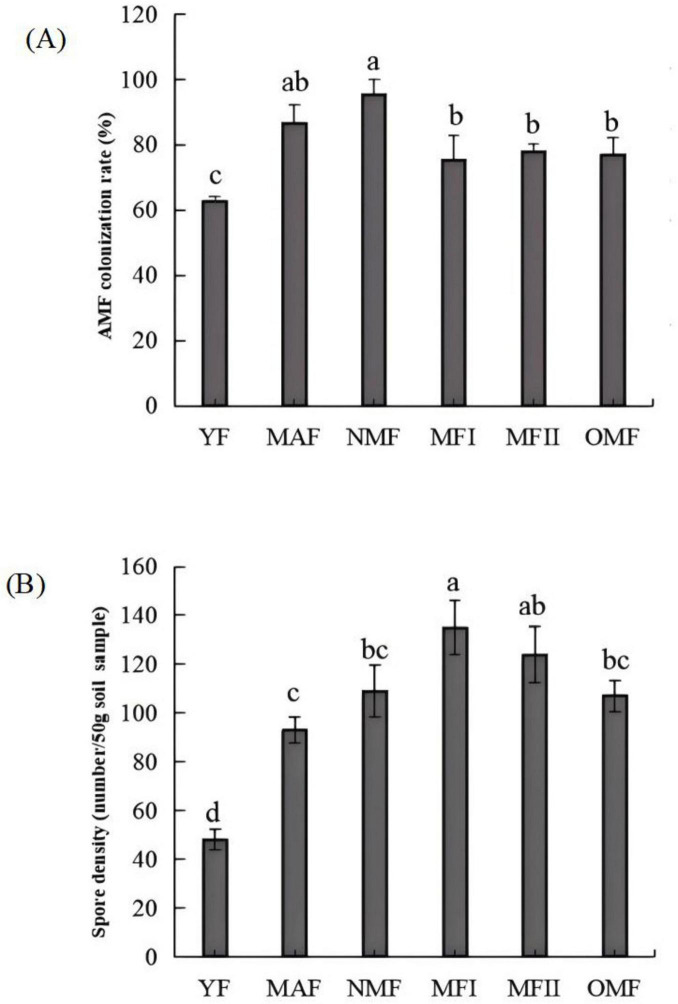
**(A)** Structure of arbuscular mycorrhizal fungi (AMF) in the root systems of *X. sorbifolium*. V, vesicles; H, hypha; Ar, arbuscular; Eh, external hyphae; **(B)** AMF spore morphotypes. (a) *Glomus multiforum*; (b) *Glomus melanosporum*; (c) *Glomus reticulatum*; (d) *Glomus constrictum*; (e) *Paraglomus occultum*; (f) *Glomus geosporum*; (g) *Claroideoglomus etunicatum*; (h) *Acaulospora lavis*; and (i) *Funneliformis mosseae*.

**FIGURE 3:**
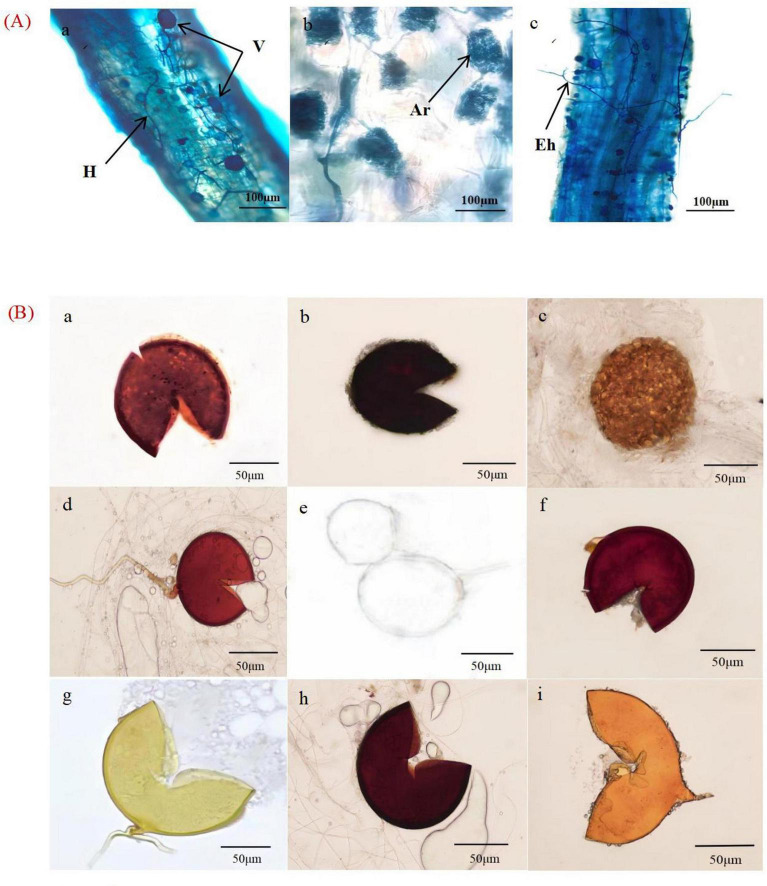
Mycorrhizal colonization rate **(A)** and spore density **(B)** of *X. sorbifolium*. Different lowercase letters indicate significant differences at *p* < 0.05. YF, 5 years; MAF, 13 years; NMF, 24 years; MFI, 35 years; MFII, 47 years; OMF, 56 years.

The AMF mycelium, vesicle and arbuscular structures were clearly visible in the root system of *X. sorbifolium* of different forest ages under the microscope, indicating that *X. sorbifolium* of different forest ages had formed a stable symbiotic relationship with AMF and formed an Arum-type arbuscular mycorrhizal (Figure 2A). The main manifestation was that the mycelium entered the plant root system and mostly grew longitudinally along the root cells (Figure 2Aa), and the lateral bifurcated arbuscular directly penetrated the cell wall of the cortex to form a typical arbuscular structure (Figure 2Ab). And during the extension of the mycelium, the end expanded and developed into the vesicles of varying sizes and diverse morphologies (Figure 2Ac). Mycorrhizal colonization rates ranged from 63 to 95%, with NMF stands having the highest colonization rates, which were significantly higher than MFI, MFII, OMF, and YF stands (*P*<0.05), but were not significantly correlated with MAF stands (*P* > 0.05) (Figure 3A).

The spore density was calculated using Equation 1. The spore density first increased and then decreased with the increase in plantation age. The spore density was calculated according to Equation 1. A total of 48–135 spores were collected from the rhizosphere soil of *X. sorbifolium* at different stand ages (Figure 1B). Spore density increased and then decreased with forest age, and MFI stands had the highest spore density, which was significantly higher than that of YF, MAF, NMF, and OMF (*P* < 0.05), but was not significantly correlated with the age of MFII stands (*P* > 0.05). 18 species of spores, belonging to 7 genera of AMF were identified by spore morphological methods. The relative abundance, separation frequency and importance value of spores were calculated by Equations 2–4. The results showed that Glomus was the most abundant and was the dominant genus. *Glomus multiforum* and *Glomus melanosporum* were detected in different forest stands and were the dominant species in the site (Supplementary Table 6). Figure 2B shows pictures of spores with high importance values in the *X. sorbifolium* AMF.

### 3.2 Abundance and diversity of AMF in *Xanthoceras sorbifolium*

Bioinformatic analysis identified 483,488 sequences from the 24 soil samples, with 82,837 for YF, 76,987 for NMF, 71,565 for MAF, 75,711 for MFI, 88,856 for MFII, and 87,532 for OMF. Similarity clustering based on the 97% queue value yielded 4,523 AMF ASVs, and the number of total ASVs was 47 (Supplementary Figure 1).

**FIGURE 4:**
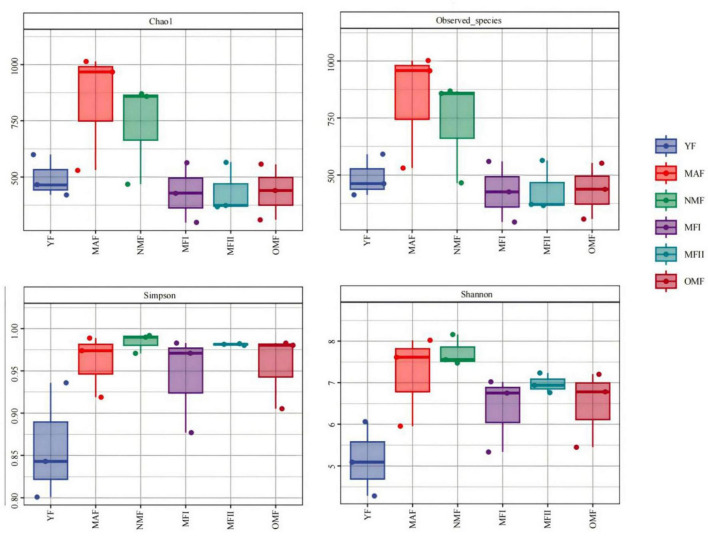
Alpha diversity of *X. sorbifolium*. YF, 5 years; MAF, 13 years; NMF, 24 years; MFI, 35 years; MFII, 47 years; OMF, 56 years.

AMF community diversity varied substantially among different stand ages, with similar trends in the Chao1 and observed_species indexes. The highest index values were observed in the MAF, while significant differences were not observed among the MFI, MFII, and OMF stands. The Shannon and Simpson diversity indexes reached their highest values in the NMF and lowest values in the YF (Figure 4).

### 3.3 AMF compositions and structures

**FIGURE 5:**
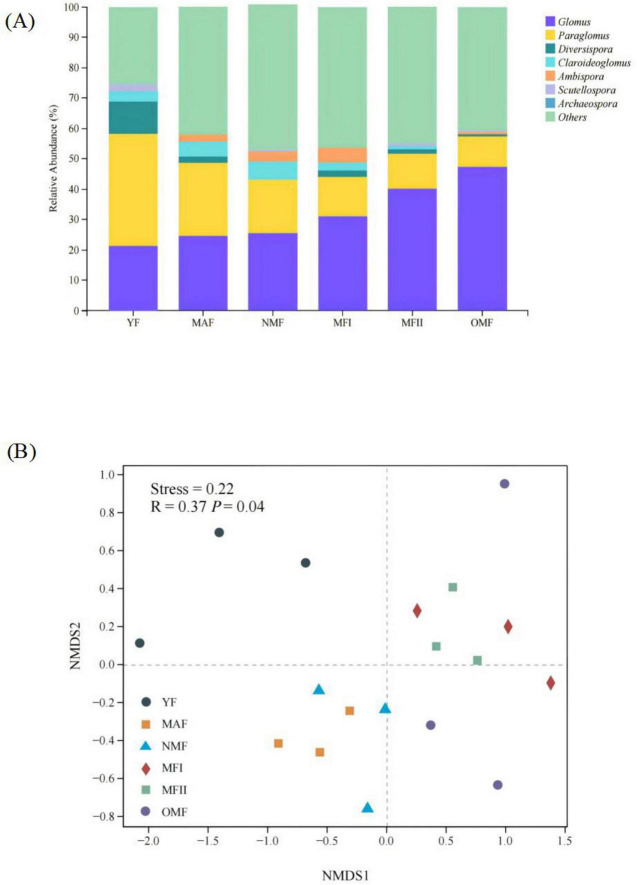
**(A)** Relative abundance of arbuscular mycorrhizal fungi (AMF) genera in *X. sorbifolium*. **(B)** Non-metric multi-dimensional scaling (NMDS) of the AMF communities associated with *X. sorbifolium*. YF, 5 years; MAF, 13 years; NMF, 24 years; MFI, 35 years; MFII, 47 years; OMF, 56 years.

The obtained ASVs belonged to 1 phylum, 4 classes, 6 orders, 12 families, and 15 genera, and the AMF community was dominated by *Paraglomus* and *Glomus*, which presented relative abundances ranging from 15.13 to 47.3% and 12.03 to 58.74%, respectively (Figure 5A). Bray-Curtis based NMDS analyses found significant differences in soil microbial communities between stand ages (*R* = 0.37, *p* = 0.04) (Figure 5A). Soil AMF communities were closer in MAF and NMF stands, and MFI, MFII, and OMF stands, indicating higher community similarity (Figure 5B).

### 3.4 Environmental variables driving AMF composition and community structure

Correlation analysis showed that the soil characteristic variables were strongly correlated with the AMF genus. Ten of the indicators were correlated with soil AMF genus (Table 1). Among them, *Glomus* was significantly and positively correlated with soil SOC, TN, AP, pH (*P* < 0.05) and highly significantly and positively correlated with soil N:P (*P* < 0.01). However, *Paraglomus* showed highly significant negative correlation with SOC, TN and TP (*P* < 0.01), and significant negative correlation with AP and pH (*P* < 0.05). *Diversispora* showed highly significant negative correlation with TN and TP (*P* < 0.01);significant positive correlation with NH_4_^+^, BD, and soil C: N (*P* < 0.05), and highly significant positive correlation with soil C: P showed highly significant positive correlation (*P* < 0.01). A total of 10 variables in plant traits were correlated with AMF genus. Among them, root-related variables were more strongly correlated with AMF genus than leaf variables. Only 6 of the litter traits (ULB, SLB, ULC, ULP, SL C:N, and SL C:P) were correlated with AMF genus. Among them, all four variables, ULB, SLB, ULC, and SL C:N, were significantly and positively correlated with *Glomu*s (*P* < 0.05). *Diversispora* was significantly negatively correlated with ULB (*P* < 0.05) and highly significantly negatively correlated with SLB (*P* < 0.01); *Scytellospora* was significantly negatively correlated with ULB, ULC, and ULP (*P* < 0.05), highly significantly positively correlated with SL C:N (*P* < 0.01), and significantly positively correlated with SL C:P (*P* < 0.05).

**TABLE 1:** Spearman’s correlation analysis between the relative abundance of AMF genera and soil, plant, and litter variables.

		*Glomus*	*Paraglomus*	*Diversispora*	*Claroideoglomus*	*Ambispora*	*Scutellospora*	*Archaeospora*
Soil	SOC	0.463*	−0.602**	−0.518	0.130	−0.026	0.137	0.024
	TN	0.483*	−0.655**	−0.864**	0.085	0.257	−0.110	−0.046
	NH_4_^+^	−0.143	0.101	0.530*	−0.139	−0.058	0.026	0.009
	BD	−0.227	0.298	0.509*	0.211	−0.045	0.400	0.162
	AP	0.475*	−0.472*	−0.420	0.117	0.460	−0.049	−0.073
	pH	0.487*	−0.476*	−0.427	−0.105	0.432	−0.242	−0.264
	TP	0.211	−0.475**	−0.764**	0.015	0.162	−0.201	−0.216
	Soil C:N	−0.334	0.441	0.898*	−0.138	−0.183	0.393	0.208
	Soil C:P	0.131	0.123	0.600**	−0.211	−0.407	−0.402	0.414
	Soil N:P	0.614**	−0.352	−0.181	−0.240	−0.314	0.083	0.242
Plant	RL	0.114	0.050	0.067	0.042	0.077	0.791**	−0.146
	SRL	0.103	0.047	0.040	0.040	−0.209	0.807**	0.006
	RSA	−0.486*	0.605**	0.737**	0.115	0.026	0.239	0.187
	RB	−0.487*	0.571*	0.093	0.107	−0.386	0.040	0.204
	RP	−0.484*	0.354	0.486*	0.289	−0.368	−0.140	0.033
	Root N:P	0.491*	−0.505*	−0.483*	−0.221	0.117	−0.175	0.187
	LC	0.541*	−0.382	−0.190	−0.146	−0.340	−0.134	0.140
	LB	0.213	−0.278	−0.453	−0.124	−0.040	−0.497*	0.185
Litter	ULB	0.481*	−0.356	−0.566*	0.110	0.016	−0.511*	−0.248
	SLB	0.567*	−0.254	−0.599**	0.290	−0.231	−0.211	−0.283
	ULC	0.451*	−0.168	−0.278	−0.008	−0.146	−0.525*	−0.076
	ULP	0.032	−0.151	−0.300	0.003	−0.122	−0.538*	−0.073
	SL C:N	0.448*	0.002	0.020	0.073	−0.057	0.800**	−0.020
	SL C:P	−0.029	0.048	0.017	0.123	−0.025	0.522*	−0.087

*, **Significant at *P* < 0.05 and *P* < 0.01, respectively.

**FIGURE 6:**
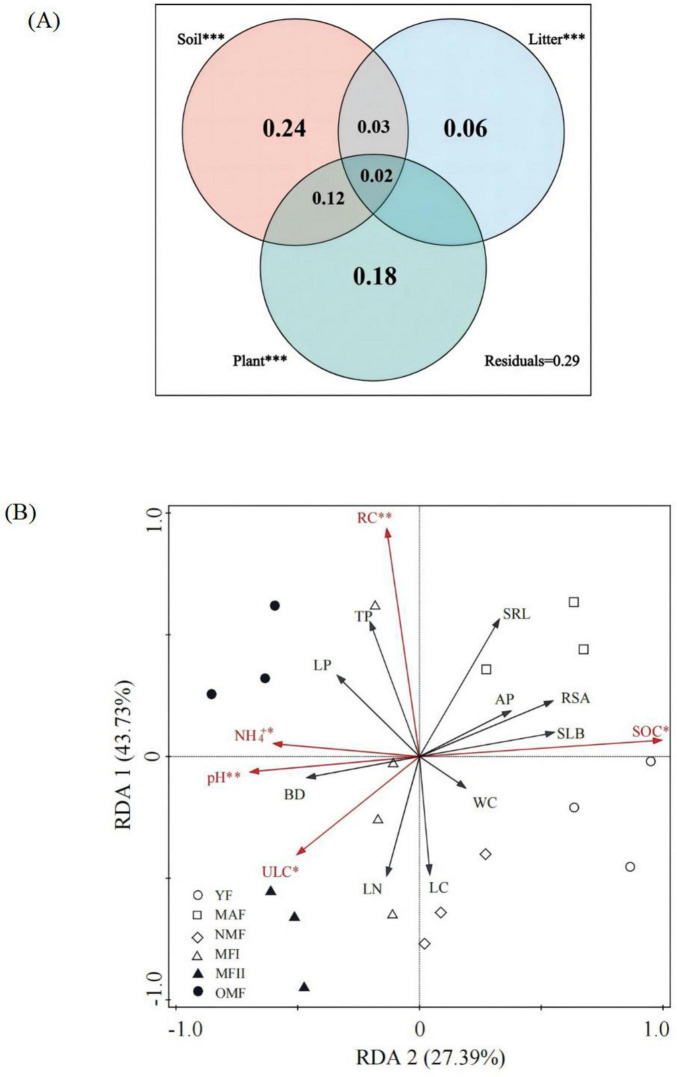
**(A)** VPA describes the proportion of AMF community variation explained by three sets of predictors: soil nutrients, plant and litter traits. Each shaded section indicates the individual contributions of the identified factors to the changes in soil properties, and the overlapping circles indicate common effects. Explained variance scores are corrected *R*^2^-values. **(B)** RDA of the relationship between Bray-Curtis dissimilarity of AMF communities and variables selected for VPA results. *, **Significant at *P*< 0.05 and *P*< 0.01, respectively. RC, Root carbon; SRL, Specific root length; RSA, Root surface area; AP, available phosphorus; SLB, semi-decomposed litter biomass; SOC, soil organic carbon; WC, water content; LC, leaf carbon; LN, leaf nitrogen; ULC, Undecomposed litter C; BD, bulk density; NH4^+^: NH4^+^-N; LP, Leaf phosphorus; TP, total phosphorus. YF, 5 years; MAF, 13 years; NMF, 24 years; MFI, 35 years; MFII, 47 years; OMF, 56 years.

The VPA results showed that soil, plant, and litter variables together explained 60% of the variation in AMF communities, and the selected variables are listed in Supplementary Table 7 (Figure 6A). The pure effects of soil, litter, and plant traits were 24, 6, and 13%, respectively. Soil properties and plant traits together explained 12% of the variation in AMF communities, and soil and litter traits together accounted for 3% of the variation in AMF. Soil, plant, and litter traits all explained highly significant of the variance in AMF communities (*P* < 0.001) (Table 2).

**TABLE 2:** Variance analysis of the AMF community characteristics explained by soil, plant, and litter characteristics.

Soil					Plant				Litter			
	**R^2^**	**Adjusted R^2^**	**F**	**Pr (>F)**	**R^2^**	**Adjusted R^2^**	**F**	**Pr (>F)**	**R^2^**	**Adjusted R^2^**	**F**	**Pr (>F)**
AMF	0.22	0.19	5.13	0.001***	0.32	0.27	2.95	0.001***	0.24	0.16	1.24	0.001***

The values of R^2^ and Adj. R^2^ indicated the proportion and adjust proportion of variance explained by the fitting models for the response variables.

*, **, ***Significant at *P* < 0.05, *P* < 0.01 and *P* < 0.001 respectively.

RDA showed that the first axis explained 43.73% and the second axis explained 27.39%, with the two axes together explaining 71.12% of the total variance (Figure 6B). Monte Carlo tests further indicated that soil pH, NH_4_^+^, and SOC, plant RC and SRL, and litter ULC were significant indicators and major contributors affecting the AMF communities (Table 3).

**TABLE 3:** RDA of the variables and AMF compositions in soil, plant, and litter samples.

	Variable	Explains (%)	Contribution (%)	Adjusted R^2^	*P*-value
Soil	pH	18.2	26.5	0.34	0.007**
	NH_4_^+^	5.9	8.8	0.28	0.026*
	SOC	5.2	7.6	0.26	0.042*
	WC	2.3	3.1	0.21	0.137
	TN	3.7	4.3	0.19	0.248
	BD	1.9	2.7	0.16	0.659
	TP	0.8	1.1	0.13	0.647
	AP	1.2	1.5	0.31	0.553
Plant	RC	15.3	22.7	0.32	0.010**
	SRL	4.0	5.2	0.25	0.046*
	RSA	1.6	2.4	0.19	0.416
	LC	0.9	1.2	0.13	0.664
	LN	1.1	1.4	0.10	0.583
	LP	1.3	1.9	0.22	0.431
Litter	ULC	5.3	8.7	0.27	0.039*
	SLB	0.6	0.8	0.16	0.643

*, **Significant at *P* < 0.05 and *P* < 0.01, respectively.

## 4 Discussion

This study presents the first comprehensive analysis of the AMF in *X. sorbifolium* plantations in Inner Mongolia, China. The results indicate that *X. sorbifolium* root systems form Arum-type structures (Figure 2A), with all showing high colonization rates (Figure 3A), indicating a robust symbiotic relationship with the AMF. In this experiment, a total of 7 genera and 18 species of AMF were identified from the rhizosphere soil of *X. sorbifolium* by morphological identification (Supplementary Table 6). Morphological identification of AMF has obvious limitations due to the complexity of their morphological characteristics, the fact that some physiological indicators and morphological features are susceptible to change with the developmental stages and habitat conditions, and the fact that some AMF do not produce spores at all at certain times of the year (Redecker et al., 2003). In order to improve the scientific validity of the morphological identification results, the diversity of *X. sorbifolium* AMF was further analyzed using Illumina MiSeq sequencing techniques, which yielded a total of 51 species in 7 genera after annotation. However, studies using a combination of traditional and molecular methods will further elucidate our uncertain understanding of plant-associated mycorrhizal genera. The results of the present study were the same for both identifications, with *Glomus* being the dominant genus, a result that is also consistent with the majority of studies, where *Glomus* has shown a high level of adaptability in different habitat environments in symbiosis with different host plants (Bonfim et al., 2016; Coutinho et al., 2015).

With the development of the stand, *Glomus* and *Paraglomus* maintained a high abundance (Figure 5A). This is partly related to the fact that they have high spore production and a unique reproductive strategy that allows them to reproduce directly through mycelium and mycorrhizae and partly due to their high resistance to adverse environments and ability to adapt to highly cyclical and disturbed environments (Gu et al., 2022; Fall et al., 2022). This finding is in line with Marinho et al. (2004), who suggested that dominant taxa are more capable of adapting to new and constantly changing environments. In the present study, *Glomus* abundance was found to increase gradually with the number of years of cultivation, which may be related to the growth and reproduction characteristics of this genus of AMF. *Glomus* easily survives and spreads by mycelium, mycospores or fragments, and they are more resistant and resilient to ecological disturbances. Consequently, *Glomus* colonization became more stable and its abundance gradually increased with years of cultivation. However, *Paraglomus* abundance and *Glomus* abundance presented opposite change trends and gradually decreased as the forest age increased, which was due to the competitive relationship between *Paraglomus* abundance and *Glomus* (Michael et al., 2010).

The results of this experiment showed that AMF α-diversity in *X. sorbifolium* did not change regularly with increasing stand age but showed an overall increasing and then decreasing trend (Figure 4). This is consistent with the results of previous studies on *Pinus massoniana* plantations (Pan et al., 2021). However, Dong et al. (2021) pointed out that the AMF diversity index decreased and then increased with the development of *Pinus massoniana*, while Gao et al. (2023) showed that the diversity index of Chinese fir plantations gradually increased with the age of the forest. Consistent conclusions have not been reached about the change rule of AMF community diversity in forest soil with stand age, indicating that the successional pattern of AMF communities is very complex and difficult to predict based on the long-term development of the forest stand (Robin et al., 2019). Changes in AMF community diversity due to different stand ages are not only influenced by metabolic activities and reproduction, but also by soil physicochemical properties, plant, and litter characteristics. In addition, changes in soil enzyme activities can affect AMF growth and diversity. Studies have shown that soil enzyme activity may decrease with increasing stand age, which may indirectly affect AMF diversity (Li et al., 2024).

The VPA results were consistent with our second hypothesis that AMF community variation with stand development was due to the combined effects of soil, plant, and litter characteristics. Of these, the soil variable independently accounted for the largest proportion of the total variance in AMF community composition, suggesting that soil properties had a significant effect on soil AMF composition (Table 1) and represented the most important driver of AMF communities (Figure 6A). Substantial evidence has shown that soil properties are the main drivers of AMF community structure and diversity (Cusack et al., 2011; Liu et al., 2020). Of the soil variables, pH, NH_4_^+^, and SOC were identified as important parameters affecting AMF communities (Figure 6B and Table 3). Soil pH, as a key soil property, is extremely important in the shaping of AMF communities in natural and plantation forest ecosystems, and soil pH ultimately affects AMF colonization in host plants and AMF communities by influencing AMF extraradical mycelial growth, spore density, and abundance (Adenan et al., 2021; Ma et al., 2021). The results of this study showed that pH is significantly positively correlated with *Glomus* and significantly negatively correlated with *Paraglomus* (Supplementary Table 8). The results also suggested that *Glomus* is more effective at colonizing neutral and alkaline soils, whereas the production of *Paraglomus* is associated with acidic soils. These results are consistent with those of Jiang et al. (2020), who showed that different AMF taxa have different preferences for soil pH. However, Yang (2022) reported that pH was positively correlated with AMF abundance, which is inconsistent with the results of our study showing that soil pH was significantly negatively correlated with α-diversity (Supplementary Table 8). These contrasting results are likely related to the differences in soil acidity, as the soil samples analyzed by Yang (2022) were alkaline, whereas the soil samples analyzed in this study were neutral or weakly acidic. Plants can directly utilize NO_3_^–^ and NH_4_^+^, which are mainly produced by decomposition of soil microorganisms decomposition, and AMF plays a very important role in the nitrogen cycle because they can directly take up and transfer NH_4_^+^ and NO_3_^–^ in the soil, which is the basis for maintaining the nitrogen balance of the ecosystem (Hodge and Storer, 2015). In this study, NH_4_^+^ had a significant effect on the AMF community while NO_3_^–^ had no significant effect. The reason may be that compared AMF absorbed NH_4_^+^ faster than NO_3_^–^ and the cost of NH_4_^+^ uptake and assimilation by AMF extraradical mycelia was less than that of NO_3_^–^ (Wei et al., 2016). Because NH_4_^+^ is more readily emitted by NH_3_ and N_2_O, this may affect the survival and activities of AMF, which in turn affects its symbiotic relationship with plants (Li S. J. et al., 2020). SOC was significantly and positively correlated with AMF diversity in the present study, which is consistent with the results of the study on the effects of geographic distance on AMF fungal communities in fruit trees (Jiang et al., 2018). Most scholars believe that an increase in soil SOC will promote AMF to decompose more organic compounds, which can improve the water retention capacity and increase nutrient supply of the soil, and further have a direct positive effect on the AMF community (Jiang et al., 2020). In this study, soil SOC was also found to be significantly positively correlated with the dominant genus of *X. sorbifolium*, *Glomus*, which taxonomically belongs to *Glomerales*. In addition, soil AMF under this order establish symbiotic relationships with host plants through vesicles and tend to use the vesicles to store lipids and SOC as energy (Brands et al., 2018). The study by Souza and Freitas (2017) similarly demonstrated the strong association of *Glomerales* with SOC.

In this study, plant traits were also identified as important drivers of AMF communities (Figure 6A), with root C and SRL identified as important influencing factors (Figure 6B and Table 3). Soil substrate is the basic condition for plant growth, and the necessary elements and nutrients required to support plant growth originate from root uptake in the soil, and plant root traits and root nutrients may also drive AMF communities. C input from fine roots is a major input to soil organic carbon stocks (McCormack et al., 2015), and C constitutes the basic structure of plants, accounting for approximately 50% of plant biomass. When soil nutrients are limiting factors, host plants usually trade large amounts of their own carbon to mycorrhizal symbionts, allowing more mycorrhizal fungal partners to compete for carbohydrates thereby increasing nutrient uptake benefits. Root C had a significant effect on AMF community structure in *X. sorbifolium*, which may be functionally related to the density of the root tissues (Wang et al., 2016), with the lower the density of root tissues the lower their activity and nutrient The lower the root tissue density the higher the activity and nutrient uptake capacity and the higher the nitrogen content and lower the carbon content. The growth of AMF requires a carbon source and other nutrients, so it significantly affects the AMF community structure. SRL, a functional strategy representing resource acquisition strategy, is an important indicator of the efficiency of nutrient uptake by fine roots. A significant relationship between root morphological traits (SRL and RSA) and AMF composition and diversity was found in this study (Table 1 and Supplementary Table 8), which is consistent with a previous study (Prada-Salcedo et al., 2021). Higher SRL and RSA provides more space for AMF survival. The fine root is where AMF exchanges material with the host plant, and AMF is more sensitive to changes in fine root traits. On the other hand, it may be because SRL and RSA can directly reflect the survival strategy of the species and the soil environmental conditions. In addition, the morphological characteristics of the root system are directly related to the nutrient uptake and carbon allocation strategy of the plant, which determines the quantity and quality of root litter and secretion and influences the changes of the AMF community. Plant characteristics are not the main driver of differences in AMF diversity. Plant investment in AMF may be lower due to changes in soil fertility and tree nutrient status along the stand age gradient. In addition, among plant leaf traits, only the leaf biomass and leaf C content were significantly correlated with AMF composition (Table 1), and AMF diversity was correlated with leaf biomass (Supplementary Table 8). Our findings are similar to the results of Li M. Y. et al. (2020), who showed that the low correlation between leaf traits and AMF composition and diversity was due to the indirect effect of leaf traits on AMF communities and revealed that material exchange between the two was subordinate to a large and complex interaction system.

Litter is the material basis of natural ecosystems, and its decomposition is a key process of nutrient cycling, which plays an important role in productivity improvement in natural ecosystems (Fang et al., 2020). In forest ecosystems, the majority of available nutrients are concentrated in the litter layer, which is not directly accessible to plant roots. Mycorrhizal fungi are crucial for releasing nutrients from the litter, improving nutrient uptake by plants, and promoting changes in loamy nutrient content. Although AMF has no known saprophytic capacity and relies on plants for carbohydrates (Smith et al., 2011), Kong et al. (2018) found that AMF mycelia proliferate in decomposing organic matter. In addition, Hodge et al. (2001) found that AMF favor the colonization of soils with added plant litter rather than host plants alone, suggesting that litter may represent a potential source of carbon. Mei et al. (2021) concluded that AMF can alleviate nutrient limitation in soil microorganisms and positively influence litter decomposition. The results of this study showed that AMF communities were correlated with undecomposed litter C content and certain litter traits were significantly correlated with the relative abundance of *Glomus* (Table 1). *Glomus* colonizes and proliferates in leaf litter and is commonly found in a variety of ecosystems. The mycelium of AMF in litter acquires litter-bound nutrients and releases the nutrients to the associated host plants as well as nearby soil microbes (Bunn et al., 2019). We found that litter N:P was strongly correlated with AMF diversity (Supplementary Table 8), which is because litter inputs can differentially regulate N and P use efficiency across the stand age gradient, thereby affecting AMF communities.

## 5 Conclusion

Our results showed that *X. sorbifolium* formed a good symbiotic relationship with AMF in different stand ages and formed Arum-type arbuscular mycorrhizal. Both Glomus and Paraglomus were dominant genera in different stand ages, Glomus gradually increased with stand age, and Paraglomus gradually decreased with stand age. Chao1, Shannon, Simpson, and Observed_species indices showed a tendency to first increase and then decrease with stand age. AMF community changes were jointly influenced by soil, plant and litter traits, and soil traits had a greater influence on AMF communities than plant and litter traits. Among them, soil (SOC, pH, NH4 +), root (SRL, C), and litter ULC variables were important factors affecting AMF communities. These results suggest that future management practices for *X. sorbifolium* plantation forests should consider the unique responses of AMF communities to soil properties, litter and plant traits. In the future, we should further investigate the driving mechanisms behind rhizosphere soil nutrient, litter, and plant-AMF community interactions in *X. sorbifolium*, which is crucial for developing more targeted and sustainable management strategies for *X. sorbifolium* plantations.

## Data Availability

Raw data have been deposited to National Center for Biotechnology Information (NCBI) under the BioProject number PRJNA1253386. https://www.ncbi.nlm.nih.gov/.

## Footnotes

1
http://invam.wvu.edu/the-fungi/species-descriptions
