# Supplementary material for: Temporal changes in arbuscular mycorrhizal fungi communities and their driving factors in Xanthoceras sorbifolium plantations
Source: Front Microbiol. 2025 May 29;16:1579868. doi: 10.3389/fmicb.2025.1579868 (PMC12158962; doi:10.3389/fmicb.2025.1579868)
Supplement: Supplementary file 1 [file Table_1.docx]

Supplementary Material

**Sampling method**

Three sample plots measuring 20 m × 20 m were established in each stand, with five small sample squares of 1.0 m × 1.0 m randomly placed within each plot. Large debris, such as dead branches and gravel, was removed from the sample squares, and litter (including leaves, branches, flowers, etc.) was collected layer by layer. The undecomposed layer was considered intact, while the semi-decomposed layer showed signs of microbial decomposition, such as surface discoloration and increased humidity. Approximately 500 g of healthy mature leaves were collected from the outer layers of the canopy of each *X. sorbifolium* tree at four cardinal directions (south-east, north-west, north-west, and south-east), respectively, from intact branches free of pests and diseases using high pruning shears, and the leaves were placed in self-sealing bags and stored in an ice box. Root samples were collected using the root drill method (Wei et al.; 2024) at a distance of 50 cm from the main stem of *X. sorbifolium*, with 20 cm sampling intervals. The roots were separated from the drill using an iron sieve, and the root samples were placed in envelope bags and stored at 4°C to prevent wilting. Plant and animal debris were removed from the soil surface, and a 0-30 cm soil profile was excavated within 30 cm of the main stem. Fine roots, close to the main root system, were collected along with approximately 1 mm thick soil attached to fine roots (≤ 2 mm in diameter), which was classified as rhizosphere soil (Edwards et al., 2015) and collected using a sterile brush. Samples from each plot were thoroughly mixed, and a total of 18 samples (litter in undecomposed and semi-decomposed layers, leaves, roots, and rhizosphere soil) were collected, representing six forest ages (YF, NMF, MAF, MFI, MFII, and OMF), with three replicates for each.

**Morphological and chemical analyses of leaves, litter, and roots**

Fine roots with a diameter < 2 mm were selected, cleaned with distilled water, and analyzed using a root scanner (EPSON TWAIN PRO [32 bit], Canada). A root morphology and structural analysis system (WINRhizo) was used to determine root length and surface area. The specific root length was defined as the ratio of the length of the root system to the biomass of the root system. Plant samples were dried in an oven at 60°C until a constant weight was achieved. The samples were then weighed and leaf, litter, and root biomass values were calculated. The C, N, and P contents were then determined after fine grinding to 0.15 mm using a ball mill (Wang et al., 2015). C content in leaves, litter, and roots was measured using K_2_Cr_2_O_7_ oxidation (Ren et al., 2017), and N and P content was measured using Kjeldahl and colorimetric methods after digestion in H_2_SO_4_-H_2_O_2_ (Lin et al., 2011).

**Soil physical and chemical analyses**

Soil total nitrogen (TN) was determined using an elemental analyzer (FLASHSMART, Germany); total phosphorus (TP) content was determined using the H_2_SO_4_-HClO_4_ decoction-molybdenum antimony antibiometric method (Parkinson et al., 2009); available phosphorus (AP) content was determined using the NaHCO_3_ leaching-molybdenum antimony colorimetric method (Bao, 2005); total potassium (TK) content was determined using the NaOH melting-molybdenum-antimony antimony anticolourimetric method (Bao, 2000); soil organic matter (SOC) was determined using the K_2_Cr_2_O_7_ oxidation method (Walkley et al., 1934); soil ammonium nitrogen (NH_4_^+^-N) and nitrate nitrogen (NO_3_^−^-N) were determined using a continuous flow analyzer (Skalar San++, The Netherlands) by leaching the soil with 2 mol·L^-1^ KCl; and soil pH was determined using the potentiometric method (Mettler FE28, China), with a 2.5:1 soil-water ratio. Soil pH was determined using a potentiometric method (Mettler FE28, China) with a water-soil ratio of 2.5:1, soil bulk density (BD) was determined using the ring knife method, and soil water content (WC) was determined using the drying method.

**References**

1. Bao, S.D. Soil agrochemical analysis. Beijing: China Agricultural Press.2000
2. Edwards, J., Johnson, C., Santos-Medellín, C., Lurie, E., Podishetty, N.K., Bhatnagar, S., Eisen,J.A., Sundaresan, V. Structure, variation, and assembly of the root-associated microbiomes of rice.Proceedings of the National Academy of Sciences. 2015, 112(8):E911–E920. https://doi.org/10. 1073/pnas.1414592112
3. Lin, C.F.; Yang, Y.S.; Guo, J.F., Chen, G.S., Xie, J.S. Fine root decomposition of evergreen broadleaved and coniferous tree species in mid-subtropical China: dynamics of dry mass, nutrient and organic fractions. Plant Soil. 2011, 338:311–327. <https://doi.org/10.1007/s11104-010-0547-3>
4. Parkinson, J.A., Allen, S.E. A wet oxidation procedure suitable for the determination of nitrogen and mineral nutrients in biological material. Comm. Soil Sci. Plant Analy. 2009, 6:1–11. <https://doi.org/10.1080/00103627509366539>
5. Ren, C.J., Chen, J., Deng, J., Zhao, F.Z., Han, X.H., Yang, G.H., Tong, X.G., Feng, Y.Z., Shelton, S., Ren, G.X. Response of microbial diversity to C:N:P stoichiometry in fine root and microbial biomass following afforestation. Biol. Fertil. Soils. 2017, 53:457–468. <https://doi.org/10.1007/s00374-017-1197-x>
6. Walkley, A.J., Black, I.A. An examination of the Degtjareff method for determining soil organic matter, and a proposed modification of the chromic acid titration method. Soil Sci. 1934.
7. Wang, W., Wang, C., Sardans, J., Tong, C., Jia, R., Zeng, C., Penuelas, J. Flood regime affects soil stoichiometry and the distribution of the invasive plants in sub- tropical estuarine wetlands in China. Catena. 2015, 128: 144–154. <https://doi.org/10.1016/j.catena.2015.01.017>
8. Wei, Z.Y., Zhang, J.J., Lai, Z. R., H, Y.W., Zhao, J.C., Tang, P., Wang, S.Q. Influence of density and site on fine root characteristics of *Pinus tabuliformis* plantations in loess area of western Shanxi Province, northern China. Journal of Beijing Forestry University2024, v. 46(10): 22-32.

Table S1. Sampling sites.

| Stand age | Tree height (m) | Diameter at breast height (cm) | Tree crown diameter (m) | Planting density (N/ha) |
| --- | --- | --- | --- | --- |
| YF | 1.38 ± 0.05c | 6.71 ± 0.35d | 3.06 ± 0.17c | 805 ± 43b |
| NMF | 3.55 ± 0.14bc | 10.25 ± 1.12c | 5.03 ± 0.29c | 930 ± 43a |
| MFI | 4.05 ± 0.26b | 13.13 ± 0.59bc | 8.92 ± 0.63b | 892 ± 64a |
| MFII | 4.80 ± 0.17a | 17.48 ± 0.69b | 11.72 ± 0.29b | 850 ± 57b |
| OMF | 5.17 ± 0.14a | 21.08 ± 0.76a | 15.03 ± 0.16a | 702 ± 46c |

Data are expressed as the mean ± standard deviation. YF, young forest; MAF, middle-aged forest; NMF, near-mature forest; MFI, mature forest I; MFII, mature forest II; OMF, over mature forest.

Table S2. *Xanthoceras sorbifolium* soil, plant, and litter variables and abbreviations

| **Soil** |  |
| --- | --- |
| Soil organic carbon | SOC |
| Total nitrogen | TN |
| Total phosphorus | TP |
| Available phosphorus | AP |
| Total potassium | TK |
| Soil C:N | Soil C:N |
| Soil C:P | Soil C:P |
| Soil N:P | Soil N:P |
| NO_3_^-^-N | NO_3_^-^ |
| NH_4_^+^-N | NH_4_^+^ |
| pH | pH |
| Bulk density | BD |
| Water content | WC |
| Plant |  |
| Leaf biomass | LB |
| Leaf carbon | LC |
| Leaf nitrogen | LN |
| Leaf phosphorus | LP |
| Leaf C:N | Leaf C:N |
| Leaf C:P | Leaf C:P |
| Leaf N:P | Leaf N:P |
| Root biomass | RB |
| Root length | RL |
| Specific root length | SRL |
| Root surface area | RSA |
| Root carbon | RC |
| Root nitrogen | RN |
| Root phosphorus | RP |
| Root C:N | Root C:N |
| Root C:P | Root C:P |
| Root N:P | Root N:P |
| Litter |  |
| Undecomposed litter biomass | ULB |
| Semi-decomposed litter biomass | SLB |
| Undecomposed litter C | ULC |
| Undecomposed litter N | ULN |
| Undecomposed litter P | ULP |
| Undecomposed litter C:N | UL C:N |
| Undecomposed litter C:P | UL C:P |
| Undecomposed litter N:P | UL N:P |
| Semi-decomposed litter C | SLC |
| Semi-decomposed litter N | SLN |
| Semi-decomposed litter P | SLP |
| Semi-decomposed litter C:N | SL C:N |
| Semi-decomposed litter C:P | SL C:P |
| Semi-decomposed litter N:P | SL N:P |

Table S3. Temporal physical and chemical properties of *Xanthoceras sorbifolium* soils.

| **Stand growth stage** | **YF** | **MAF** | **NMF** | **MFI** | **MFII** | **OMF** |
| --- | --- | --- | --- | --- | --- | --- |
| **SOC (g/kg)** | 5.98 ± 0.29d | 6.63 ± 0.24cd | 9.21 ± 0.28a | 8.34 ± 0.29b | 10.22 ± 0.13a | 7.43 ± 0.05c |
| **TN (g/kg)** | 0.46 ± 0.08d | 0.81 ± 0.06c | 1.08 ± 0.04a | 0.93 ± 0.01b | 0.95 ± 0.01b | 0.90 ± 0.02ab |
| **TP (g/kg)** | 0.12 ± 0.02e | 0.20 ± 0.02bc | 0.33 ± 0.02a | 0.27 ± 0.02b | 0.21 ± 0.01c | 0.17 ± 0.01d |
| **AP (mg/kg)** | 4.87 ± 1.11b | 9.60 ± 1.49ab | 10.43 ± 2.52a | 10.24 ± 0.19a | 10.31 ± 0.15a | 10.33 ± 0.05a |
| **TK (g/kg)** | 3.21 ± 0.03b | 3.19 ± 0.01b | 2.88 ± 0.04c | 2.90 ± 0.25c | 3.54 ± 0.08a | 3.55 ± 0.04a |
| **Soil C:N** | 13.28 ± 1.32a | 8.25 ± 1.02c | 8.48 ± 0.92c | 8.92 ± 0.87b | 10.83 ± 1.54b | 8.45 ± 0.56c |
| **Soil C:P** | 49.98 ± 4.65a | 33.43 ± 3.54b | 27.61 ± 2.78c | 30.78 ± 4.54b | 48.73 ± 2.67a | 43.82 ± 3.53a |
| **Soil N:P** | 3.88 ± 0.32bc | 4.24 ± 0.54b | 3.36 ± 0.54d | 3.67 ± 0.65c | 4.41 ± 0.34b | 5.56 ± 0.78a |
| **NO_3_^-^ (mg/kg)** | 4.54 ± 0.12a | 4.34 ± 0.19ab | 4.53 ± 0.15a | 4.21 ± 0.20b | 4.01 ± 0.09c | 4.39 ± 0.35ab |
| **NH_4_^+^ (mg/kg)** | 15.08±1.23a | 8.07±1.01c | 8.01±1.21c | 11.98±0.95b | 10.01±1.02b | 12.45±1.17ab |
| **pH** | 7.10 ± 0.05a | 7.14 ± 0.05a | 7.17 ± 0.13a | 7.16 ± 0.05a | 7.13 ± 0.03a | 7.15 ± 0.06a |
| **BD (g/cm^3^)** | 1.32 ± 0.03a | 1.28 ± 0.02ab | 1.30 ± 0.02a | 1.13 ± 0.06ab | 1.04 ± 0.04c | 0.99 ± 0.08d |
| **WC (%)** | 10.78 ± 0.33 | 12.56 ± 0.40 | 11.91 ± 0.49 | 11.78 ± 1.03 | 10.54 ± 0.59 | 10.21 ± 0.20 |

Data are expressed as mean ± standard deviation. Different lower-case letters indicate significant differences between stand ages (P < 0.05). YF, young forest; MAF, middle-aged forest; NMF, near-mature forest; MFI, mature forest I; MFII, mature forest II; OMF, over mature forest.

**Table S4.** Temporal leaf and root traits of *Xanthoceras sorbifolium*.

| **Stand growth stage** | **MAF** | **NMF** | **NMF** | **MFI** | **MFII** | **OMF** |
| --- | --- | --- | --- | --- | --- | --- |
| **LB (t/ha)** | 2.65 ± 0.09d | 2.77 ± 0.05d | 3.54 ± 0.05c | 4.06 ± 0.07b | 4.64 ± 0.22ab | 5.03 ± 0.07a |
| **LC (g/kg)** | 464.67 ± 8.91c | 474.22 ± 10.39b | 487.78 ± 23.11a | 491.88 ± 18.77a | 472.22 ± 10.3b | 479.66 ± 45.08ab |
| **LN (g/kg)** | 33.73 ± 4.94b | 34.82 ± 0.71ab | 37.75 ± 3.05a | 36.83 ± 2.16a | 32.66 ± 2.36b | 29.36 ± 3.53c |
| **LP (g/kg)** | 2.09 ± 0.11d | 2.28 ± 0.08c | 2.31 ± 0.13c | 2.74 ± 0.20a | 2.54 ± 0.07b | 2.54 ± 0.07b |
| **Leaf C:N** | 13.64 ± 0.93c | 13.54 ± 1.03c | 13.32 ± 0.89c | 13.43 ± 0.98c | 14.43 ± 1.23b | 16.15 ± 1.45a |
| **Leaf C:P** | 214.1 ± 8.23a | 210.45 ± 7.23a | 210.54 ± 7.67a | 176.54 ± 9.89b | 185.42 ± 13.98b | 184.67 ± 9.43b |
| **Leaf N:P** | 15.21 ± 1.78a | 15.01 ± 1.34a | 16.65 ± 1.45a | 12.98 ± 1.67b | 12.79 ± 1.89b | 11.67 ± 1.32b |
| **RB (g/m^2^)** | 129.34 ± 45.98a | 115.54 ± 75.04a | 95.90 ± 18.33b | 87.93 ± 17.41bc | 70.34 ± 36.21c | 64.32 ± 16.42c |
| **RL (m/m^2^)** | 226.95 ± 73.31a | 102.45 ± 37.19b | 80.94 ± 15.57c | 68.43 ± 8.04d | 104.31 ± 29.96b | 71.23 ± 26.78d |
| **SRL (m/g)** | 2.17 ± 1.25a | 1.11 ± 0.42b | 0.85 ± 0.14c | 0.81 ± 0.18c | 1.48 ± 0.66ab | 1.18 ± 0.43b |
| **RSA (m/m^2^)** | 0.85 ± 0.20ab | 0.93 ± 0.21a | 0.70 ± 0.18b | 0.54 ± 0.18c | 0.33 ± 0.07d | 0.29 ± 0.08e |
| **RC (mg/g)** | 465.69 ± 31.09a | 455.23 ± 21.17a | 431.76 ± 10.36b | 444.26 ± 22.68ab | 453.39 ± 25.09a | 468.58 ± 23.68a |
| **RN (mg/g)** | 8.64 ± 0.07c | 8.93 ± 0.24b | 12.56 ± 0.57a | 11.34 ± 0.72a | 10.39 ± 0.62ab | 8.76 ± 0.15c |
| **RP (mg/g)** | 1.28 ± 0.01a | 1.08 ± 0.01b | 1.04 ± 0.01b | 0.98 ± 0.03c | 1.11 ± 0.02ab | 0.85 ± 0.01d |
| **Root C:N** | 53.44 ± 3.84a | 50.83 ± 1.81a | 35.66 ± 2.31c | 40.21 ± 3.05bc | 42.98 ± 4.98bc | 52.96 ± 3.52b |
| **Root C:P** | 363.39 ± 32.90c | 420.74 ± 60.92b | 416.69 ± 226.96ab | 455.28 ± 186.53ab | 407.07 ± 13.93b | 551.40 ± 21.78a |
| **Root N:P** | 6.85 ± 0.87c | 8.26 ± 0.94b | 11.87 ± 2.81a | 11.89 ± 0.98a | 9.21 ± 1.16b | 10.31± 0.08a |

Data are expressed as mean ± standard deviation. Different lower-case letters indicate significant differences between stand ages (P < 0.05). YF, young forest; MAF, middle-aged forest; NMF, near-mature forest; MFI, mature forest I; MFII, mature forest II; OMF, over mature forest.

Table S5. Temporal litter traits of *Xanthoceras sorbifolium*.

| **Stand growth stage** | **YF** | **MAF** | **NMF** | **MFI** | **MFII** | **OMF** |
| --- | --- | --- | --- | --- | --- | --- |
| **ULB (t/hm^2^)** | 2.11 ± 0.11c | 2.35 ± 0.09b | 2.47 ± 0.16b | 3.23 ± 0.07a | 3.00 ± 0.37a | 3.18 ± 0.18a |
| **SLB (t/hm^2^)** | 2.31 ± 0.16c | 2.79 ± 0.33b | 3.19 ± 0.32a | 3.21 ± 0.11a | 3.21± 0.16a | 3.23 ± 0.26a |
| **ULC (g/kg)** | 421.33 ± 9.86b | 437.55 ± 30.93ab | 442.96 ± 19.79a | 458.55 ± 29.52a | 455.22 ± 20.31a | 462.99 ± 25.19a |
| **ULN (g/kg)** | 18.95 ± 1.10c | 23.57 ± 1.75b | 26.46 ± 1.91ab | 28.13 ± 1.13a | 30.65 ± 2.22a | 26.67 ± 0.81ab |
| **ULP (g/kg)** | 2.25 ± 0.13d | 2.54 ± 0.10c | 2.89 ± 0.25b | 2.92 ± 0.15ab | 3.32 ± 0.20a | 3.25 ± 0.13a |
| **UL C:P** | 187.59 ± 19.82a | 173.58 ± 22.87a | 152.94 ± 19.73b | 150.34 ± 18.45b | 136.78 ± 25.43c | 149.99 ± 20.44b |
| **UL N:P** | 8.37 ± 1.25c | 9.12 ± 1.54ab | 9.18 ± 1.64ab | 9.65 ± 1.57a | 9.31 ± 1.88a | 8.87 ± 0.78b |
| **SLC (g/kg)** | 321.56 ± 9.94b | 335.24 ± 25.38ab | 343.51 ± 24.22ab | 356.33 ± 29.49a | 352.32 ± 20.13a | 361.1 ± 29.47a |
| **SLN (g/kg)** | 18.04 ± 1.50c | 22.76 ± 1.95b | 25.20 ± 1.82ab | 26.91 ± 0.98ab | 28.39 ± 1.90a | 25.82 ± 0.17ab |
| **SLP (g/kg)** | 2.29 ± 0.13d | 2.64 ± 0.11c | 2.97 ± 0.23bc | 3.06 ± 0.14b | 3.51 ± 0.23a | 3.19 ± 0.14b |
| **SL C:N** | 17.99 ± 0.47a | 14.82 ± 1.47b | 13.75 ± 1.83b | 13.22 ± 0.63bc | 12.44 ± 0.81c | 13.98 ± 1.08b |
| **SL C:P** | 140.77 ± 7.48a | 127.53 ± 12.44b | 116.94 ± 16.68b | 116.47 ± 10.31b | 100.43 ± 0.87c | 113.13 ± 7.13b |
| **SL N:P** | 7.89 ± 0.76c | 8.62 ± 0.54a | 8.49 ± 0.17ab | 8.79 ± 0.49a | 8.11 ± 0.56b | 8.11 ± 0.35b |

Data are expressed as mean ± standard deviation. Different lower-case letters indicate significant differences between stand ages (P < 0.05). YF, young forest; MAF, middle-aged forest; NMF, near-mature forest; MFI, mature forest I; MFII, mature forest II; OMF, over mature forest.

Table S6. AMF species and distributions in *Xanthoceras sorbifolium* sample data.

| **Genera** | **Species** | **Different stand ages** | | | | | | **Separation**  **frequency (F, %)** | **Relative**  **abundance**  **(Ra, %)** | **Importance**  **Value**  **(Iv, %)** |
| --- | --- | --- | --- | --- | --- | --- | --- | --- | --- | --- |
|  |  | **YF** | **MAF** | **NMF** | **MFI** | **MFII** | **OMF** |  |  |  |
| *Glomus* | *geosporum* | + | + | － | － | + | + | 66.67 | 5.02 | 35.845 |
|  | *hyderabadensis* | － | + | + | － | － | － | 33.33 | 1.99 | 17.66 |
|  | *melanosporum* | + | + | + | + | + | + | 100.00 | 10.17 | 55.085 |
|  | *reticulatum* | － | + | + | + | － | + | 66.67 | 4.34 | 35.505 |
|  | *versiforme* | － | － | + | － | － | + | 33.33 | 2.11 | 17.72 |
|  | *flavisporum* | + | － | + | － | － | － | 33.33 | 2.31 | 17.82 |
|  | *multiforum* | + | + | + | + | + | + | 100.00 | 11.21 | 55.605 |
|  | *constrictum* | + | + | + | － | － | + | 66.67 | 5.75 | 36.21 |
| *Paraglomus* | *occultum* | － | － | + | － | － | + | 33.33 | 1.99 | 17.66 |
| *Claroideoglomus* | *lamellosum* | － | + | + | + | + | － | 66.67 | 3.75 | 35.21 |
|  | *etunicatum* | + | + | + | + | + | － | 83.33 | 6.15 | 44.74 |
| *Ambispora* | *jimgerdemannii* | － | + | － | － | － | + | 33.33 | 2.54 | 17.935 |
| *Acaulospora* | *lavis* | + | － | + | － | + | + | 66.67 | 4.89 | 35.78 |
|  | *excavata* | － | + | + | + | － | － | 50.00 | 3.45 | 26.725 |
|  | *foveata* | + | + | － | － | + | + | 66.67 | 3.68 | 35.175 |
| *Scutellospora* | *nigra* | － | + | + | － | － | － | 33.33 | 2.46 | 17.895 |
|  | *scytata* |  | + |  |  |  |  | 16.67 | 1.19 | 8.93 |
| *Funneliformis* | *mosseae* | － | － | + | + | + | + | 66.67 | 5.59 | 36.13 |

“+” means AMF species present in soil of the stands, “-” means AMF does not exist.

**
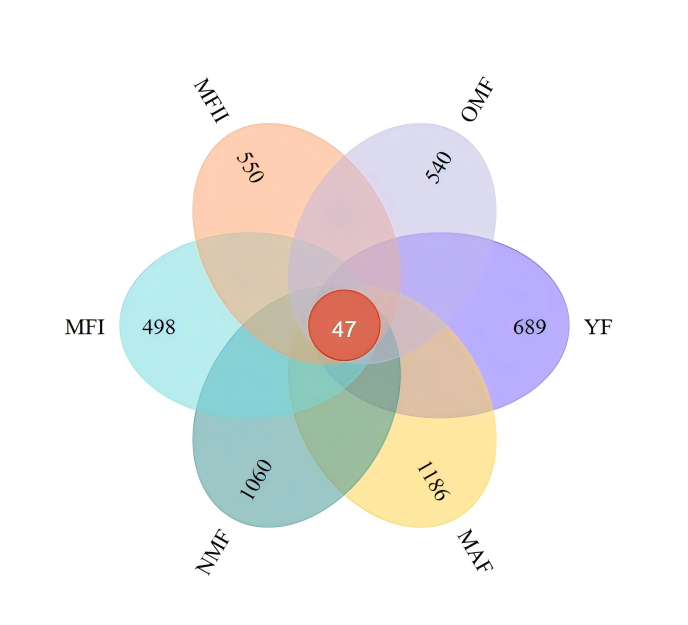
**

Fig. S1 Venn diagram representing the number of arbuscular mycorrhizal fungi (AMF) Amplicon sequence variants (ASVs) that were unique or shared between the six site.

Table S7. Selection of variables explaining variance in AMF communities through forward modelling.

| **Explanatory sets** | **Variables** | **AIC** | **F** | **P** | **VIF** |
| --- | --- | --- | --- | --- | --- |
| Soil | NH_4_^+^ | −2.746 | 1.220 | 0.045* | 7.10 |
|  | pH | −3.054 | 1.518 | 0.005** | 2.32 |
|  | BD | −3.460 | 1.921 | 0.005** | 9.58 |
|  | WC | −2.860 | 1.332 | 0.050** | 6.84 |
|  | TN | −3.322 | 1.495 | 0.005** | 2.28 |
|  | SOC | −3.012 | 1.378 | 0.005** | 2.38 |
|  | TP | −2.960 | 1.352 | 0.050** | 5.84 |
|  | AP | −3.382 | 1.445 | 0.005** | 3.28 |
| Plant | SRL | −3.010 | 1.135 | 0.030* | 4.81 |
|  | RSA | −3.231 | 1.215 | 0.005** | 5.16 |
|  | RC | −3.453 | 1.282 | 0.025* | 3.53 |
|  | LC | −2.945 | 1.325 | 0.005** | 2.06 |
|  | LN | −2.684 | 1.100 | 0.255 | 5.72 |
|  | LP | −2.774 | 1.176 | 0.150 | 4.92 |
| Litter | ULC | −3.528 | 1.988 | 0.005** | 8.61 |
|  | SLB | −2.976 | 1.181 | 0.070* | 9.88 |

*, ** indicate significant at P<0.05, P<0.01 levels, respectively.

Table S8. Correlation analysis of AMF diversity index with soil, plant and litter variables.

| **Variables** | | **Chao 1** | **Observed_Species** | **Shannon** | **Simpson** |
| --- | --- | --- | --- | --- | --- |
| Soil | NH_4_^+^ | -0.08 | −0.283 | -0.483* | -0.471* |
|  | TN | 0.035 | 0.519* | 0.602** | 0.662** |
|  | SOC | -0.314 | 0.509* | 0.293 | 0.431 |
|  | TP | 0.228 | 0.290 | 0.553* | 0.500* |
|  | AP | 0.301 | 0.272 | 0.626** | 0.622** |
|  | pH | 0.683** | −0.462 | 0.484* | 0.281 |
|  | Soil C:N | -0.346 | -0.079 | -0.596** | -0.576* |
| Plant | SRL | -0.471* | 0.512* | -0.007 | 0.246 |
|  | RSA | 0.551* | -0.464 | 0.072 | -0.191 |
|  | Root N:P | 0.280 | 0.020 | 0.519* | 0.370 |
|  | Root N | 0.051 | 0.057 | 0.496* | 0.397 |
|  | Root C | -0.481* | 0.457 | 0.234 | 0.240 |
|  | LB | -0.451 | 0.502* | -0.005 | 0.240 |
| Litter | ULC | 0.212 | -0.090 | 0.550* | 0.557* |
|  | SLB | 0.163 | -0.074 | 0.535* | 0.557* |

*, ** indicate significant at P<0.05, P<0.01 levels, respectively.
